# Association between overall dietary quality and constipation in American adults: a cross-sectional study

**DOI:** 10.1186/s12889-022-14360-w

**Published:** 2022-10-27

**Authors:** Qingye Liu, Yulong Kang, Jin Yan

**Affiliations:** 1 Department of Anus-intestines, Changzhou Wujin Hospital of Traditional Chinese Medicine, 213161 Changzhou, P.R. China; 2 Department of Proctology, Subei People’s Hospital of Jiangsu Province, No.98 Nantong Western Road, Guangling District, 225001 Yangzhou, P.R. China

**Keywords:** Healthy eating index, Overall dietary quality, Constipation, Stool consistency, Stool frequency

## Abstract

**Background:**

Constipation seriously affects people’s life quality, and dietary adjustment has been one of the effective methods. Overall dietary quality has been reported to be associated with some diseases, while its association with constipation has not been reported. This study aims to explore the association between overall dietary quality and constipation.

**Methods:**

A cross-sectional study was designed and data were extracted from National Health and Nutrition Examination Survey (NHANES). Overall dietary quality was assessed by healthy eating index-2015 (HEI-2015), and constipation was defined by either stool consistency or stool frequency. The association between overall dietary quality or components of HEI-2015 and constipation was assessed using logistic regression, with results expressed as odds ratio (OR) and 95% confidence intervals (95%CI). Subgroup analysis was conducted according to age and gender.

**Results:**

A total of 13,945 participants were eligible, with 1,407 in constipation group and 12,538 in non-constipation group. Results showed that higher adherence to HEI-2015 was associated with reduced odds of constipation (OR: 0.98, 95%CI: 0.98–0.99) after adjusting potential confounders. Further, we found higher intake of total fruits, whole fruits, total vegetables, greens and beans, whole grains, total protein foods, seafood and plant proteins, and higher fatty acids ratio decreased the odds of constipation, while higher intake of sodium increased the odds (all *P* < 0.05). We also found negative association between HEI-2015 and constipation in participants with male sex, female sex, age ≥ 65 years, and age < 65 years (all *P* < 0.05).

**Conclusion:**

We found higher adherence to HEI-2015 decreased the odds of constipation, suggesting that increasing HEI-2015 adherence may be one of effective methods to alleviate constipation.

**Supplementary information:**

The online version contains supplementary material available at 10.1186/s12889-022-14360-w.

## Background

Constipation is characterized by bowel movements less than three times a week and hard, dry, or lumpy stools [1]. The prevalence of constipation ranges from 7 to 10% in adults, and the prevalence varies by age [2, 3]. Furthermore, the incidence of constipation is different between man and women, with 6% higher in women than that of men [4]. Constipation is associated with poor quality of life, resulting in anxiety, depression, and cognitive impairment [5, 6]. Besides, constipation elevates healthcare burden. The cost of constipation treatment is estimated over $230 million each year in America [7]. Generally, constipation is treated with dietary adjustment prior to medical intervention to minimize side effects of some drugs [8].

Healthy eating index (HEI) is a measure used to evaluate the overall dietary quality, and HEI is density-based rather than absolute amounts since the 2015 version [9]. HEI-2015 is scored based on the Dietary Guidelines for Americans (DGA) and consist of 13 food groups or nutrients with their correspondent to recommended intake levels [9]. The nine adequacy components of HEI include total fruits, whole fruits, total vegetables, greens and beans, whole grains, dairy, total protein foods, seafood and plant proteins, and fatty acids. The four moderation components include refined grains, sodium, added sugars, and saturated fats. The maximum score of each component is 5 or 10, and the total score is 100, with a higher score indicating better compliance. Studies have reported the relationships between single nutrients or foods and constipation, such as lipid-rich foods, total fats, starch, sugary products, sodium, and water intake [10, 11]. In real life, people do not consume individual nutrients or certain foods, but rather mixed foods containing various nutrients [12].

Under this background, overall dietary quality has gained attention in studies of the relationship between diet and diseases [13, 14]. Association between overall dietary quality assessed by HEI and some diseases, such as diabetes, hypertension, and sleep disorder has been elucidated [15–17]. However, studies on the association between HEI-2015 and constipation have not been reported. In view of this, our study aims to assess associations of HEI-2015 with constipation.

## Methods

### Study design and data source

This was a cross-sectional study designed to conduct a secondary data analysis based on the National Health and Nutrition Examination Survey (NHANES) database (https://www.cdc.gov/nchs/nhanes/about_nhanes.htm) and the National Center for Health Statistics (NCHS) collected the data. NHANES is a research project aimed to evaluate the health and nutritional status of adults and children in the America. Each year, the survey visits 15 of counties across the country and examines a nationally representative sample of about 5,000 persons. The unique of this database is the combination of interviews and physical examinations. The interview contains demographic, dietary, socioeconomic, and health-related questions. The examination includes medical, dental, physiological, and laboratory measurements. Written informed consent was provided by all participants at the time of the survey. All NHANES data are de-identified and all NHANES studies have been approved by the National Center for Health Statistics Research Ethics Review Board (ERB) (https://www.cdc.gov/nchs/nhanes/irba98.htm). The requirement of ethical approval was waived by the Institutional Review Board of Subei People’s Hospital of Jiangsu Province, because the data was accessed from NHANES (a publicly available database). All procedures were carried out in accordance with relevant guidelines and regulations (https://www.cdc.gov/nchs/data_access/restrictions.htm).

### Study population

The data of participants were extracted from 2005 to 2010 NHANES due to these three 2-year cycles (2005–2006, 2007–2008, 2009–2010) recorded data on constipation. Participants who aged equal to or over 20 years old, completed at least one valid 24-hour dietary recall, and with complete bowel health questionnaire were included. Participants who had extreme total energy intakes of < 500 or > 5000 kcal/day for women, and < 500 or > 8000 kcal/day for men [18], with self-reported history of inflammatory bowel disease (ulcerative colitis or crohn’s disease), celiac disease, and/or colon cancer, and pregnant women were excluded.

The 24-hour dietary recall interview were conducted by trained dietary interviewers fluent in Spanish and English. Dietary data were collected on both weekdays and weekends, and participants were requested to recall all the food and beverages consumed in the past 24 h (midnight to midnight). To obtain a more complete picture of daily dietary intake, two 24-hour dietary recalls were requested. The first dietary recall was performed by in-person interview in the Mobile Examination Center (MEC). The second dietary recall was performed by telephone scheduled 3 to 10 days later. Participants were provided with a standard set of measuring guides to help participants estimate portion sizes during both interviews, as well as a food model booklet to help report food amounts during the telephone interview [19–21].

The dietary data were collected with a computer-assisted food coding and utilized the U.S Department of Agriculture (USDA) Automated Multiple-Pass Method to account for day-to-day variations. The nutritional values of all food and beverages were provided by the USDA’s Food and Nutrient Database for Dietary Studies (FNDDS), which was regularly updated each cycle and supplied the nutrient profiles for every food and beverage reported in NHANES [19, 20, 22]. In this study, we used the reported value for the participants completed one 24-hour dietary recalls, and we used the average value for those completed two recalls [19].

### Dietary quality assessment

The dietary quality was assessed by HEI-2015, which contained nine adequacy components and four moderation components [9]. For adequacy components, they were encouraged to consume and higher scores reflected higher intakes. For moderation components, they were limited to consume and higher scores reflected lower intakes. The adequacy components were total fruits, whole fruits, total vegetables, greens and beans, whole grains, dairy, total protein foods, seafood and plant proteins, and fatty acids [ratio of poly- and monounsaturated fatty acids (PUFAs and MUFAs) to saturated fatty acids (SFAs)]. The moderation components were refined grains, sodium, added sugars, and saturated fats. Supplementary Table 1 shows the components, recommended intake levels, and calculation method of HEI-2015. The total score of HEI-2015 was 100, with higher score indicating better overall dietary quality.

### Constipation definition

Constipation was defined by either stool consistency or stool frequency based on the NHANES database [23, 24]. Stool consistency and stool frequency were recorded for 30 days before data collection.

Stool consistency was estimated using the Bristol stool form scale, which included various colorful cards and detailed descriptions of seven stool types (Type 1: separate hard lumps, like nuts; Type 2: sausage-like, but lumpy; Type 3: like a sausage but with cracks in the surface; Type 4: like a sausage or snake, smooth and soft; Type 5: soft blobs with clear-cut edges; Type 6: fluffy pieces with ragged edges, a mushy stool; and Type 7: watery, no solid pieces) [25]. Participants were asked “Please look at this card and tell me the number that corresponds with your usual or most common stool type?” Constipation was defined as Type 1 or Type 2. Types 3–7 were defined as non-constipation [25].

Stool frequency was assessed with the following question: “How many times per week do you usually have a bowel movement?” Those who answered less than 3 times per week was defined as constipation, and 3 times or over per week was defined as non-constipation.

### Data collection

The data were extracted based on demographic characteristics [gender, age, race, education level, marital status, family income, comorbidity (hypertension and depression), and body mass index (BMI)]; lifestyle characteristics (drinking, smoking, vigorous physical activity, and moderate physical activity); dietary intake (total energy intake); laboratory parameters (cotinine); and dietary supplements, calcium supplement, and use of laxatives.

The demographic characteristics (assessed via interview) included gender (male or female), age (divided into < 65 years and ≥ 65 years), race (Mexican American, Other Hispanic, Non-hispanic White, Non-Hispanic Black, Other Race-Including Multi-Racial), education level (less than or equal to 12th grade, General Educational Development, and college or above), marital status (married, widowed, divorced/separated, and never married), family income (< 20,000$ and ≥ 20,000$), and comorbidity (hypertension and depression), and BMI. BMI was not self-reported, and it was calculated as body weight (kg)/height (m)^2^. Weight and height were measured at the MEC. Weight was taken on a Toledo digital scale. When participants were properly positioned and the digital readout was stable, the recorder called the weight and entered it into the weight box of the automated system. Weight was measured in pounds and converted to kilograms in the automated system. Standing height was an evaluation of maximum vertical size, and measured with a fixed stadiometer with a vertical backboard and a moveable headboard. Participants were request to remove any ornaments and braids from the top of the head for accurate stature measurement.

The lifestyle characteristics contained drinking, smoking, vigorous physical activity, and moderate physical activity (assessed via interview). The participants had at least 12 alcohol drinks per year was defined as drinking. Those who smoked at least 100 cigarettes in the entire life was defined as smoking. The moderate physical activity was defined by doing moderate activities, such as brisk walking, bicycling for pleasure, golf, and dancing, that cause only light sweating or a slight to moderate increase in breathing or heart rate for at least 10 min over the past 30 days. The vigorous activities were defined by doing any vigorous activities, such as running, lap swimming, aerobics classes, or fast bicycling, that caused heavy sweating or large increases in breathing or heart rate for at least 10 min over the past 30 days.

Dietary supplements, calcium supplement, and use of laxatives were self-reported. When asked “Have you used or taken any vitamins, minerals or other dietary supplements in the past 30 days?”, someone answered “Yes” was considered to take dietary supplements. The intake of calcium supplement was obtained using 24-hour dietary recall.

### Statistical analysis

The data in this study were weighted using appropriate sample weights provided by NHANES to account for complex sampling design of NHANES. The continuous data were represented as mean (standard error) (S.E), and differences between groups were compared using t test. The categorical data were shown as number (n) and percentage (%), and differences between groups were examined using chi-squared test. The missing data were processed using multiple imputation through R 4.0.3 (R Foundation for Statistical Computing, Vienna, Austria).

Logistic regression analysis was used to explore the association between HEI-2015 and constipation, and results were expressed as odds ratio (OR) with 95% confidence intervals (95%CI). Model 1 was unadjusted model. Model 2 adjusted age and gender to decrease the selective bias. Based on Model 2, Model 3 further adjusted race, education level, marital status, family income, BMI, drinking, smoking, vigorous physical activity, moderate physical activity, hypertension, depression, total energy intake, dietary supplements and calcium supplement which had statistical significance in general characteristics between the two groups.

The multiplicative interaction between age (or gender) and HEI-2015 on the odds of constipation was assessed using the logistic regression analysis. The sensitivity analysis was performed to avoid the bias caused by multiple imputation and different definitions of constipation. The subgroup analysis was performed based on age and gender. All statistical analyses were performed using SAS 9.4 (SAS Institute Inc., Cary, NC, USA) and two-sided *P* < 0.05 was considered statistically significant.

## Results

### Participants selection and general characteristics of the selected participants

**Fig. 1:**
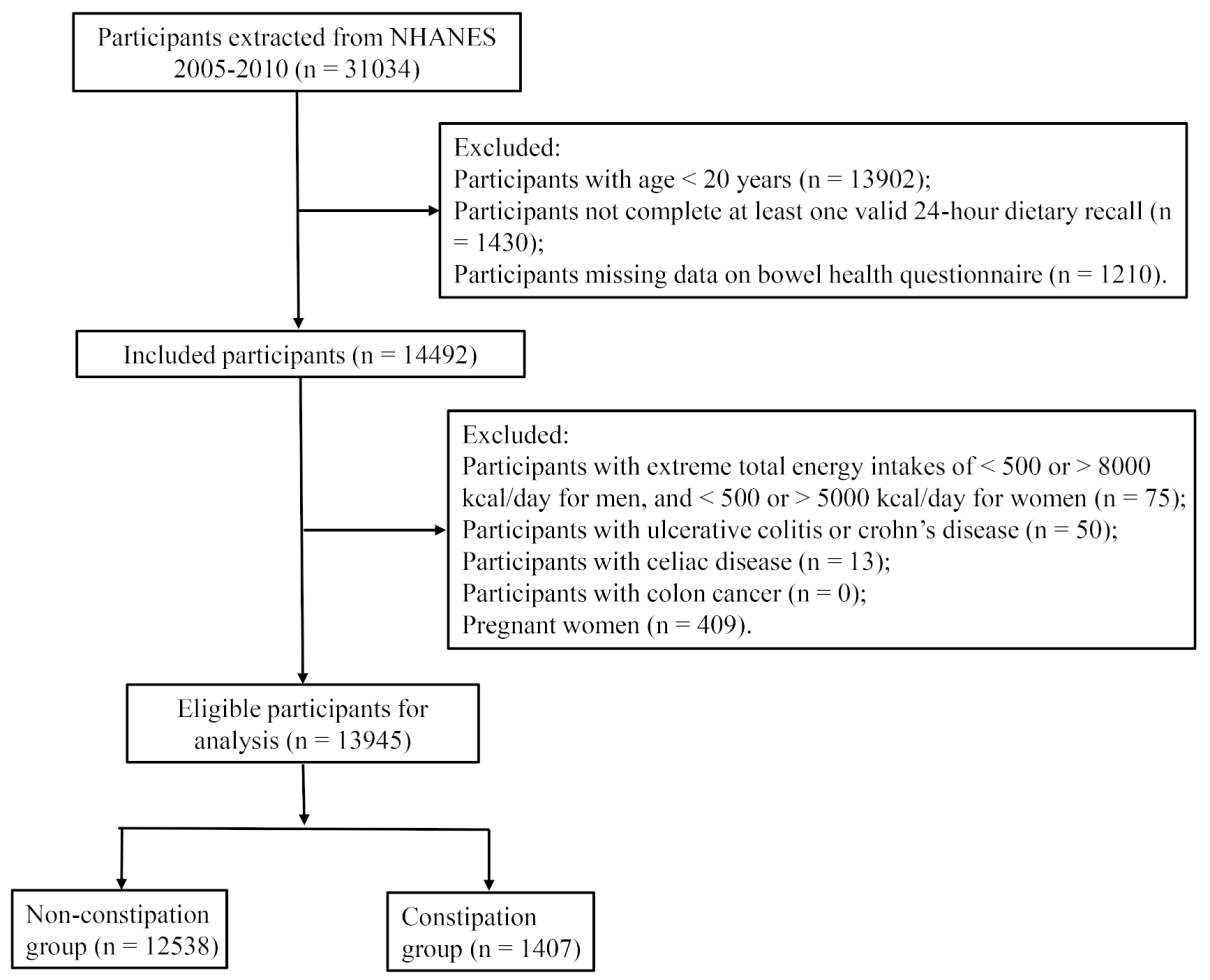
The flowchart of selecting participants

We extracted 31,034 participants from the NHANES database. After excluding participants with age < 20 years (n = 13,902), not complete at least one valid 24-hour dietary recall (n = 1,430), and missing data on bowel health questionnaire (n = 1,210), 14,492 participants were included. Further, we excluded participants with extreme total energy intakes (n = 75), with ulcerative colitis or crohn’s disease (n = 50), with celiac disease (n = 13), with colon cancer (n = 0), and pregnant women (n = 409), 13,945 participants were eligible for analysis (1,407 participants in the constipation group and 12,538 participants in the non-constipation group) (Fig. 1).

Of the eligible participants, there were 49.59% of male (n = 7,075) and 82.68% of participants aged less than 65 years (n = 10,488). Gender, BMI, race, education level, marital status, family income, drinking, smoking, vigorous physical activity, moderate physical activity, hypertension, depression, total energy intake, dietary supplements, calcium supplement, and HEI score were significantly different between constipation group and non-constipation group (all *P* < 0.05) (Table 1). Further, we compared the components of HEI-2015 between the two groups, and found the statistical difference in whole fruits, total vegetables, greens and beans, whole grains, total protein foods, seafood and plant proteins, sodium, and added sugars (Supplementary Table 2).

**Table 1:** General Characteristics of the participants from the National Health and Nutrition Examination Survey 2005–2010

**Characteristic**	**Total** **(n = 13,945)**	**Non-constipation (n = 12,538)**	**Constipation** **(n = 1407)**	***P***
Gender, n (%)				< 0.001
Female	6870 (50.41)	5898 (47.96)	972 (73.85)	
Male	7075 (49.59)	6640 (52.04)	435 (26.15)	
Age, n (%)				0.202
< 65 years	10,488 (82.68)	9395 (82.54)	1093 (84.05)	
≥ 65 years	3457 (17.32)	3143 (17.46)	314 (15.95)	
BMI, kg/m^2^, Mean (S.E)	28.71 (0.10)	28.80 (0.11)	27.84 (0.25)	< 0.001
Race, n (%)				< 0.001
Mexican American	2506 (8.04)	2280 (8.07)	226 (7.80)	
Non-Hispanic Black	2779 (10.84)	2411 (10.28)	368 (16.21)	
Non-Hispanic White	6938 (71.45)	6314 (72.02)	624 (65.96)	
Other Hispanic	1171 (4.33)	1030 (4.24)	141 (5.20)	
Other Race	551 (5.33)	503 (5.39)	48 (4.83)	
Education level, n (%)				< 0.001
≤ 12th	3906 (18.15)	3447 (17.71)	459 (22.32)	
GED	3361 (24.53)	2971 (24.02)	390 (29.49)	
College or above	6678 (57.32)	6120 (58.27)	558 (48.19)	
Marital status, n (%)				< 0.001
Married	7433 (57.15)	6774 (57.83)	659 (50.60)	
Widowed	1193 (5.89)	1055 (5.69)	138 (7.75)	
Divorced/separated	1982 (12.68)	1763 (12.47)	219 (14.71)	
Never married	3330 (24.28)	2939 (24.00)	391 (26.94)	
Family Income, n (%)				< 0.001
< 20,000$	3476 (17.43)	3022 (16.73)	454 (24.18)	
≥ 20,000$	10,469 (82.57)	9516 (83.27)	953 (75.82)	
Drinking, n (%)				< 0.001
No	3915 (24.02)	3380 (22.93)	535 (34.46)	
Yes	10,030 (75.98)	9158 (77.07)	872 (65.54)	
Smoking, n (%)				< 0.001
No	7265 (52.55)	6456 (52.03)	809 (57.56)	
Yes	6680 (47.45)	6082 (47.97)	598 (42.44)	
Vigorous physical activity, n (%)				0.007
No	10,763 (71.62)	9627 (71.08)	1136 (76.78)	
Yes	3182 (28.38)	2911 (28.92)	271 (23.22)	
Moderate physical activity, n (%)				< 0.001
No	8010 (50.27)	7133 (49.79)	877 (54.87)	
Yes	5935 (49.73)	5405 (50.21)	530 (45.13)	
Hypertension, n (%)				0.015
No	9012 (69.14)	8047 (68.84)	965 (71.96)	
Yes	4933 (30.86)	4491 (31.16)	442 (28.04)	
Depression, n (%)				< 0.001
No	12,751 (92.99)	11,558 (93.71)	1193 (86.02)	
Yes	1194 (7.01)	980 (6.29)	214 (13.98)	
Cotinine, ng/mL, Mean (S.E)	62.56 (2.58)	62.35 (2.55)	64.66 (5.72)	0.657
Total energy intake, kcal, Mean (S.E)	2129.06 (13.61)	2153.76 (14.76)	1891.98 (20.16)	< 0.001
Dietary supplements, n (%)				< 0.001
No	7248 (48.33)	6444 (47.76)	804 (53.86)	
Yes	6697 (51.67)	6094 (52.24)	603 (46.14)	
Calcium supplement (mg), Mean (S.E)	957.87 (9.03)	970.38 (9.34)	837.81 (15.51)	< 0.001
Use of laxatives, n (%)				0.117
No	13,891 (99.66)	12,493 (99.70)	1398 (99.34)	
Yes	54 (0.34)	45 (0.30)	9 (0.66)	
HEI score, Mean (S.E)	52.94 (0.29)	53.19 (0.29)	50.54 (0.43)	< 0.001

Abbreviation: BMI, body mass index; S.E, standard error; GED, General Educational Development; HEI, healthy eating index

Note: The continuous data were shown as mean (S.E), and differences between groups were compared using t test. The categorical data were shown as number and percentage [n (%)], and differences between groups were compared using chi-squared test

### Association between HEI-2015 score and constipation

To explore the association between HEI-2015 score and constipation, logistic regression analysis was used. In the unadjusted model, HEI-2015 was negatively associated with constipation (OR: 0.98, 95%CI: 0.98–0.99). Similar result was observed after adjusting age and gender (OR: 0.98, 95%CI: 0.98–0.99). Further adjusting race, education level, marital status, family income, BMI, drinking, smoking, vigorous physical activity, moderate physical activity, hypertension, depression, total energy intake, dietary supplements, and calcium supplement, higher HEI-2015 score was associated with the lower odds of constipation. The score was increased by 1 point, and the odds was reduced by 2% (OR: 0.98, 95%CI: 0.98–0.99) (Table 2). To avoid bias caused by multiple imputation, we performed the sensitivity analysis focused on the association of HEI-2015 with constipation before imputation. The results were consistent with those after imputation (Supplementary Table 3).

**Table 2:** Association between overall dietary quality and constipation

**Variables**	**Model 1**		**Mode 2**		**Model 3**	
	**OR (95%CI)**	***P***	**OR (95%CI)**	***P***	**OR (95%CI)**	***P***
HEI score	0.98 (0.98–0.99)	< 0.001	0.98 (0.98–0.99)	< 0.001	0.98 (0.98–0.99)	< 0.001

Abbreviation: OR, odds ratio; CI, confidence interval; HEI, healthy eating index

Model 1, unadjusted model;

Model 2, adjusted for age and gender;

Model 3, adjusted for age, gender, race, education level, marital status, family income, BMI, drinking, smoking, vigorous physical activity, moderate physical activity, hypertension, depression, total energy intake, dietary supplements, and calcium supplement

Further, we explored the association between components of HEI-2015 and constipation. Results showed that higher intake of total fruits (OR: 0.96, 95%CI: 0.93–0.99), whole fruits (OR: 0.96, 95%CI: 0.92–0.99), total vegetables (OR: 0.89, 95%CI: 0.85–0.93), greens and beans (OR: 0.96, 95%CI: 0.92–0.99), whole grains (OR: 0.96, 95%CI: 0.93–0.99), total protein foods (OR: 0.90, 95%CI: 0.85–0.97), seafood and plant proteins (OR: 0.96, 95%CI: 0.93–0.99), higher fatty acids ratio (OR: 0.97, 95%CI: 0.95–0.99) decreased the odds of constipation, while higher intake of sodium (OR: 1.02, 95%CI: 1.01–1.04) increased the odds in fully adjusted model (Table 3).

**Table 3:** Association between components of HEI-2015 and constipation

**Variables**	**Model 1**		**Mode 2**		**Model 3**	
	**OR (95%CI)**	***P***	**OR (95%CI)**	***P***	**OR (95%CI)**	***P***
Total fruits	0.97 (0.94-1.00)	0.063	0.95 (0.92–0.98)	0.003	0.96 (0.93–0.99)	0.042
Whole fruits	0.96 (0.93–0.99)	0.012	0.94 (0.91–0.97)	< 0.001	0.96 (0.92–0.99)	0.022
Total vegetables	0.89 (0.86–0.93)	< 0.001	0.86 (0.83–0.90)	< 0.001	0.89 (0.85–0.93)	< 0.001
Greens and beans	0.95 (0.91–0.98)	0.006	0.93 (0.90–0.97)	< 0.001	0.96 (0.92–0.99)	0.028
Whole grains	0.96 (0.93–0.98)	0.002	0.95 (0.92–0.97)	< 0.001	0.96 (0.93–0.99)	0.005
Dairy	1.00 (0.97–1.02)	0.690	0.98 (0.96–0.99)	0.045	1.03 (0.99–1.06)	0.108
Total protein foods	0.85 (0.80–0.91)	< 0.001	0.89 (0.83–0.95)	< 0.001	0.90 (0.85–0.97)	0.003
Seafood and plant proteins	0.94 (0.91–0.96)	< 0.001	0.94 (0.91–0.96)	< 0.001	0.96 (0.93–0.99)	0.018
Fatty acids ratio	0.98 (0.96-1.00)	0.060	0.98 (0.96-1.00)	0.070	0.97 (0.95–0.99)	0.003
Refined grains	0.98 (0.96-1.00)	0.070	0.98 (0.96-1.00)	0.107	0.99 (0.96–1.01)	0.190
Sodium	1.03 (1.01–1.05)	0.004	1.03 (1.01–1.05)	0.001	1.02 (1.01–1.04)	0.017
Added sugars	0.92 (0.91–0.94)	< 0.001	0.92 (0.91–0.94)	< 0.001	0.98 (0.94–1.02)	0.247
Saturated fats	1.01 (0.99–1.03)	0.447	1.01 (0.98–1.03)	0.540	0.97 (0.92–1.02)	0.211

Abbreviation: OR, odds ratio; CI, confidence interval; HEI, healthy eating index

Model 1, unadjusted model;

Model 2, adjusted for age and gender;

Model 3, adjusted for age, gender, race, education level, marital status, family income, BMI, drinking, smoking, vigorous physical activity, moderate physical activity, hypertension, depression, total energy intake, dietary supplements, and calcium supplement

In addition, we explored the association between HEI-2015 and constipation according to different definitions of constipation. Based on the stool consistency definition, one score increased in HEI-2015 decreased 1% odds of constipation (OR: 0.99, 95%CI: 0.98–0.99) in fully adjusted model. Based on the stool frequency definition, the similar outcome was found in fully adjusted model, with one score increase of HEI-2015 decreasing 3% odds of constipation (OR: 0.97, 95%CI: 0.97–0.98). The results were shown in Supplementary Table 4.

### Association between HEI-2015 score and constipation in different gender and ages

The association between HEI-2015 score and constipation was explored based on gender and ages. After adjusting age, race, education level, marital status, family income, BMI, drinking, smoking, vigorous physical activity, moderate physical activity, hypertension, depression, energy intake, dietary supplements, and calcium supplement, we found every 1-point increase in HEI-2015 score was associated with 2% decreased odds of constipation in male (OR: 0.98, 95%CI: 0.97–0.99). The similar result was observed in female, with OR of 0.98 (95%CI: 0.98–0.99). For participants with age ≥ 65 years, HEI-2015 score was significantly associated with the reduced odds of constipation (OR: 0.98, 95% CI: 0.98–0.99). A similar result was observed for participants with age < 65 years, and OR was 0.98 (95% CI: 0.98–0.99). There was no multiplicative interaction between age or gender and HEI-2015 on the odds of constipation (Table 4).

**Table 4:** Association between overall dietary quality and constipation in participants with different gender and age

	**Variables**	**Model**	
		**OR (95%CI)**	***P***
Gender ^a^			
Male	HEI score	0.98 (0.97–0.99)	0.007
Female	HEI score	0.98 (0.98–0.99)	< 0.001
Gender * HEI score			0.930
Age ^b^			
≥ 65 years	HEI score	0.98 (0.98–0.99)	< 0.001
< 65 years	HEI score	0.98 (0.98–0.99)	< 0.001
Age * HEI score			0.829

Abbreviation: OR, odds ratio; CI, confidence interval; HEI, healthy eating index

Model ^a^, adjusted for age, race, education level, marital status, family income, BMI, drinking, smoking, vigorous physical activity, moderate physical activity, hypertension, depression, energy intake, dietary supplements, and calcium supplement

Model ^b^, adjusted for gender, race, education level, marital status, family income, BMI, drinking, smoking, vigorous physical activity, moderate physical activity, hypertension, depression, total energy intake, dietary supplements, and calcium supplement

## Discussion

In this study, we explored whether overall dietary quality was associated with constipation in adults from NHANES database with a large and nationally representative sample. After adjusting a wide range of potential confounders, we found the negative association between HEI-2015 score and constipation, and this association was not affected by different definitions of constipation. We also found the components of HEI-2015 that is total fruits, whole fruits, total vegetables, greens and beans, whole grains, total protein foods, seafood and plant proteins, fatty acids ratio decreased the odds of constipation, and that is sodium increased the odds. Either in male or female, HEI-2015 score was negatively associated with constipation. Similarly, we found higher adherence to HEI-2015 was associated with lower odds of constipation in participants with age ≥ 65 years and < 65 years.

Constipation has been reported to impair the quality of life, and the estimated prevalence of constipation is approximately 10% worldwide [2, 26]. Dietary adjustment is considered as an effective strategy to alleviate or treat this symptom [8]. It is impossible to distinguish the effect of specific dietary ingredients due to the interaction of nutrients in foods. Understanding overall dietary quality and its association with constipation overcomes the challenge of assessing the individual food or nutrient and considering their interaction effects. Since 1995, HEI has been a dietary index which is developed to assess the dietary quality according to DGA [27]. By the evidence-based recommendations of USDA and Health and Human Services (HHS), HEI is updated every five years [28, 29]. The latest version is HEI-2015, pointing out two essential features in nutrition guidelines, namely, adequacy and moderation for dietary intakes, and the first part is encouraged to intake and the second part is limited to intake [9]. HEI-2015 highlighted the higher intake of fruits, vegetables, whole grains, beans, dairy, seafood and plant proteins, that is consistent with Mediterranean diet [9, 30]. Evidence has shown the significant association between higher Mediterranean diet adherence and decreased constipation syndrome scores [31, 32]. HEI-2015 limits the intake of refined grains, sodium, added sugars, and saturated fats, while Western dietary pattern is characterized by the high intake of these food items [33, 34], and has been reported to less improve constipation [35]. Accordingly, our study found that higher adherence to HEI-2015 was associated with the reduced odds of constipation. The different definitions of constipation may cause some biases, leading to the inconsistent results [25]. In our study, whether the constipation defined by stool consistency or stool frequency, we found the similar association, indicating the robustness of our results.

To further clarify our findings, we explored the association between components of HEI-2015 and constipation. The results showed the negative association between total fruits, whole fruits, total vegetables, greens and beans, whole grains, total protein foods, seafood and plant proteins, fatty acids ratio and constipation, and positive association between sodium and constipation. Yang et al. have supported that higher consumption of fruits and vegetables decreased the prevalence of constipation [3]. This may be explained by that fruits and vegetables were rich in soluble fibers, which had a high affinity for water to soft the stools, thereby helping defecate [36]. Studies have revealed that the consumption of beans could improve the constipation [37, 38]. One potential explaining for this was that oligosaccharides in beans could stimulate the development of bifidobacterium to play a laxative role [39]. Rollet et al. found higher intake of whole grains was associated with a lower constipation score [10]. Whole grains were a rich source of dietary fiber (10–12 g/100 g), which increased fecal bulk and promoted faster time of intestinal/colonic transit [10]. Evidence also indicated the negative association between higher fatty acids ratio (PUFA/SFA) and constipation [10], due to SFA could change gut microbiota composition, potentially resulting in intestinal dysbiosis, while PUFA, such as omega-3 or long-chain PUFAs, could regulate inflammation and immune system, promoting a healthy symbiosis of intestinal bacteria [10, 40]. Also, the negative relationship between plant proteins and constipation was reported by You et al. [41] In addition, sodium was found to be positively associated with the odds of constipation [10]. This may be explained by that higher intake of salt reduced the amount of water in the stool, making it difficult to move along the digestive tract and causing difficulties in defecation [42].

Constipation has been reported to be associated with gender and age [43, 44]. Constipation was common in the elderly, inadequate intake of fiber or fluid and decrease of physical activity may increase the prevalence of constipation in elderly people, with the prevalence ranging from 9 to 60% [44, 45]. In our study, we found the negative association between HEI-2015 and constipation in participants aged < 65 years or ≥ 65 years. These findings suggested that people with age of either < 65 years or ≥ 65 years should increase the adherence of HEI-2015 to prevent constipation. Female gender was also an independent risk factor for constipation, and the prevalence of constipation in females was approximately twice as likely as males [43, 46]. Our study found that higher adherence to HEI-2015 was associated with lower odds of constipation in male and female, indicating the importance of increasing HEI-2015 adherence for both male and female to decrease the odds of constipation.

Our study includes a relatively larger sample size to explore the association between overall dietary quality and constipation. To avoid bias caused by multiple imputation and different definitions of constipation, we performed sensitivity analysis, which increases the robustness of results. Further, we perform subgroup analysis to determine the association in different populations. There are some limitations in our study. First, although we have adjusted the potential confounders affecting the constipation, there are still some confounders that may not be considered due to the limited data in the database. Second, this is a cross-sectional study, which cannot address causality. In the future, a prospective study should be designed to further investigate the association between the overall dietary quality and constipation.

## Conclusion

In conclusion, we found that higher adherence to HEI-2015 was associated with the lower odds of constipation. The similar results were found in participants with age of < 65 years or ≥ 65 years and with gender of male or female. Our findings suggested that increasing HEI-2015 adherence to alleviate symptoms of constipation may be one of effective methods. Prior to medical intervention, clinicians should promote and emphasize a balanced diet as first line treatment.

## Electronic supplementary material

 The online version contains supplementary material available at https://doi. org/10.1186/s12889-022-14360-w.

Supplementary Material 1

## Data Availability

The datasets generated and/or analyzed during the current study are available in the NHANES database, https://wwwn.cdc.gov/nchs/nhanes/.

## Abbreviations

HEI: Healthy eating index
DGA: Dietary Guidelines for Americans
NHANES: National Health and Nutrition Examination Survey
ERB: Ethics Review Board
BMI: body mass index
