# Supplementary material for: Association between overall dietary quality and constipation in American adults: a cross-sectional study
Source: BMC Public Health. 2022 Oct 27;22:1971. doi: 10.1186/s12889-022-14360-w (PMC9615246; doi:10.1186/s12889-022-14360-w)
Supplement: Supplementary file 1 — Supplementary Material 1 [file 12889_2022_14360_MOESM1_ESM.docx]

**Supplementary Table 1 HEI-2015^1^ components and scoring standards**

| **Component** | **Maximum points** | **Standard for maximum score** | **Standard for minimum score of zero** |
| --- | --- | --- | --- |
| **Adequacy** |  |  |  |
| Total fruits^2^ | 5 | ≥ 0.8 cup equivalents per 1,000 kcal | No fruits |
| Whole fruits^3^ | 5 | ≥ 0.4 cup equivalents per 1,000 kcal | No whole fruits |
| Total vegetables^4^ | 5 | ≥ 1.1 cup equivalents per 1,000 kcal | No vegetables |
| Greens and beans^4^ | 5 | ≥ 0.2 cup equivalents per 1,000 kcal | No dark green vegetables or legumes |
| Whole grains | 10 | ≥ 1.5 oz equivalents per 1,000 kcal | No whole grains |
| Dairy^5^ | 10 | ≥ 1.3 cup equivalents per 1,000 kcal | No dairy |
| Total protein foods^6^ | 5 | ≥ 2.5 oz equivalents per 1,000 kcal | No protein foods |
| Seafood and plant proteins^6,7^ | 5 | ≥ 0.8 oz equivalents per 1,000 kcal | No seafood or plant proteins |
| Fatty acids^8^ | 10 | (PUFAs + MUFAs)/SFAs ≥ 2.5 | (PUFAs + MUFAs)/SFAs ≤ 1.2 |
| **Moderation** |  |  |  |
| Refined grains | 10 | ≤ 1.8 oz equivalents per 1,000 kcal | ≥ 4.3 oz equivalents per 1,000 kcal |
| Sodium | 10 | ≤ 1.1 gram per 1,000 kcal | ≥ 2.0 grams per 1,000 kcal |
| Added sugars | 10 | ≤ 6.5% of energy | ≥ 26% of energy |
| Saturated fats | 10 | ≤ 8% of energy | ≥ 16% of energy |

Abbreviation: HEI, healthy eating index.

Note:

^1^Intakes between the minimum and maximum standards are scored proportionately. The total HEI score is the sum of the adequacy components (i.e. foods to eat more of for good health) and moderation components (i.e. foods to limit for good health).

^2^Includes 100% fruit juice.

^3^Includes all forms except juice.

^4^Includes legumes (beans and peas).

^5^Includes all milk products, such as fluid milk, yogurt, and cheese, and fortified soy beverages.

^6^Includes legumes (beans and peas).

^7^Includes seafood, nuts, seeds, soy products (other than beverages), and legumes (beans and peas).

^8^Ratio of poly- and monounsaturated fatty acids (PUFAs and MUFAs) to saturated fatty acids (SFAs).

Reference: Krebs-Smith SM, Pannucci TE, Subar AF, Kirkpatrick SI, Lerman JL, Tooze JA, et al. Update of the Healthy Eating Index: HEI-2015. Journal of the Academy of Nutrition and Dietetics. 2018; 118: 1591-602.

**Supplementary Table 2 Comparison of components of HEI-2015 between constipation group and non-constipation group**

| **Characteristic** | **Total**  **(n=13945)** | **Non-constipation (n=12538)** | **Constipation**  **(n=1407)** | ***P*** |
| --- | --- | --- | --- | --- |
| Total fruits, Mean (S.E) | 2.39 (0.04) | 2.40 (0.04) | 2.29 (0.06) | 0.062 |
| Whole fruits, Mean (S.E) | 2.45 (0.04) | 2.47 (0.04) | 2.29 (0.07) | 0.011 |
| Total vegetables, Mean (S.E) | 3.30 (0.02) | 3.32 (0.02) | 3.07 (0.05) | < 0.001 |
| Greens and beans, Mean (S.E) | 1.82 (0.04) | 1.84 (0.04) | 1.60 (0.08) | 0.005 |
| Whole grains, Mean (S.E) | 2.52 (0.04) | 2.56 (0.05) | 2.20 (0.09) | 0.001 |
| Dairy, Mean (S.E) | 5.43 (0.05) | 5.43 (0.05) | 5.39 (0.11) | 0.689 |
| Total protein foods, Mean (S.E) | 4.45 (0.01) | 4.47 (0.01) | 4.29 (0.04) | < 0.001 |
| Seafood and plant proteins, Mean (S.E) | 2.73 (0.04) | 2.76 (0.04) | 2.45 (0.06) | < 0.001 |
| Fatty acids ratio, Mean (S.E) | 4.81 (0.05) | 4.83 (0.05) | 4.62 (0.11) | 0.058 |
| Refined grains, Mean (S.E) | 6.20 (0.05) | 6.22 (0.05) | 5.99 (0.13) | 0.073 |
| Sodium, Mean (S.E) | 4.24 (0.04) | 4.21 (0.04) | 4.52 (0.11) | 0.004 |
| Added sugars, Mean (S.E) | 6.58 (0.07) | 6.67 (0.06) | 5.74 (0.14) | < 0.001 |
| Saturated fats, Mean (S.E) | 6.03 (0.05) | 6.02 (0.06) | 6.11 (0.10) | 0.445 |

Abbreviation: HEI, healthy eating index; S.E, standard error.

Note: The continuous data were shown as mean (S.E), and differences between groups were compared using t test. The categorical data were shown as number and percentage [n (%)], and differences between groups were compared using chi-squared test.

**Supplementary Table 3 Association between overall dietary quality and constipation before multiple imputation**

| **Variables** | **Model 1** | | **Mode 2** | | **Model 3** | |
| --- | --- | --- | --- | --- | --- | --- |
|  | **OR (95%CI)** | ***P*** | **OR (95%CI)** | ***P*** | **OR (95%CI)** | ***P*** |
| HEI score | 0.98 (0.98-0.99) | < 0.001 | 0.98 (0.98-0.99) | < 0.001 | 0.98 (0.98-0.99) | < 0.001 |

Abbreviation: OR, odds ratio; CI, confidence interval; HEI, healthy eating index.

Model 1, unadjusted model;

Model 2, adjusted for age and gender;

Model 3, adjusted for age, gender, race, education level, marital status, family income, BMI, drinking, smoking, vigorous physical activity, moderate physical activity, hypertension, total energy intake, dietary supplements, and calcium supplement.

**Supplementary Table 4 Association between overall dietary quality and constipation defined by stool consistency and stool frequency**

| **Variables** | **Model 1** | | **Mode 2** | | **Model 3** | |
| --- | --- | --- | --- | --- | --- | --- |
|  | **OR (95%CI)** | ***P*** | **OR (95%CI)** | ***P*** | **OR (95%CI)** | ***P*** |
| HEI score ^a^ | 0.99 (0.99-0.99) | 0.002 | 0.99 (0.98-0.99) | < 0.001 | 0.99 (0.98-0.99) | 0.008 |
| HEI score ^b^ | 0.99 (0.98-0.99) | < 0.001 | 0.97 (0.96-0.97) | < 0.001 | 0.97 (0.97-0.98) | < 0.001 |

Abbreviation: OR, odds ratio; CI, confidence interval; HEI, healthy eating index.

Model 1, unadjusted model;

Model 2, adjusted for age and gender;

Model 3, adjusted for age, gender, race, education level, marital status, family income, BMI, drinking, smoking, vigorous physical activity, moderate physical activity, hypertension, total energy intake, dietary supplements, and calcium supplement.

Constipation is defined by ^a^ stool consistency and ^b^ stool frequency, respectively.
